# *Pseudouroctonus peccatum*, a new scorpion from the Spring Mountains near “Sin City,” Nevada (Scorpiones, Vaejovidae)

**DOI:** 10.3897/zookeys.364.6288

**Published:** 2013-12-18

**Authors:** Amanda E. Tate, Rebecca R. Riddle, Michael E. Soleglad, Matthew R. Graham

**Affiliations:** 1 School of Life Sciences, University of Nevada, Las Vegas, 4505 S Maryland Pkwy, Las Vegas, NV 89154 USA; 2 32255 Safflower St., Winchester CA 92596, USA; 3 Department of Biology, Eastern Connecticut State University, 83 Windham Street, Willimantic, CT 06226 USA

**Keywords:** Barcoding, COI, hemispermatophore, *Pseudouroctonus*, taxonomy, *Uroctonites*

## Abstract

A new scorpion species is described from the Spring Mountain Range near Las Vegas, Nevada. The new species appears to be geographically isolated from other closely related species of *Uroctonites* Williams & Savaryand *Pseudouroctonus* Stahnke. We tentatively place the new species in *Pseudouroctonus* and provide detailed descriptions and illustrations of type material. We compare the new species to 17 congeneric taxa, briefly discuss the taxonomic history of *Pseudouroctonus*, and provide DNA barcodes for two paratypes to assist ongoing research on the systematics of family Vaejovidae.

## Introduction

Low dispersal potential and ecological specialization (stenotopy) are thought to make certain groups of scorpions predisposed to accelerated diversification (Prendini 2005; Bryson et al. 2013a). Scorpions restricted to highland ecosystems are particularly diverse, perhaps resulting from allopatric divergence on small spatial scales (microallopatry, see Fitzpatrick et al. 2008) facilitated by historical changes in geomorphology and climate regimes (Bryson et al. 2013a, 2013b). Recently, this hypothesis has been repeatedly supported by the discovery of numerous new scorpion species from isolated mountain ecosystems, especially from the “sky islands” region of the North American aridlands (Graham 2007; Ayrey 2009, 2012; Graham and Bryson 2010; Ayrey and Soleglad 2011; Hughes 2011; Graham et al. 2012; Sissom et al. 2012; Ayrey and Webber 2013).

The Spring Mountain Range, located just outside of Las Vegas in southern Nevada, is among the most insular of the sky islands, reaching elevations more than 3,400 m above the Mojave Desert lowlands. While conducting diurnal surveys for myriapods in the Spring Mountains, we serendipitously discovered yet another new scorpion that appears to be restricted to a sky island ecosystem. After numerous diurnal and nocturnal (using UV light; Stahnke 1972) surveys, we only managed to collect a total of five individuals from mixed pine-oak woodlands in Kyle Canyon, one of the most heavily visited regions in the Spring Mountains. Unfortunately, forest fires ravaged the type locality shortly after we collected the type series and surveys in other areas of the mountains were unsuccessful.

**Figure 1.**
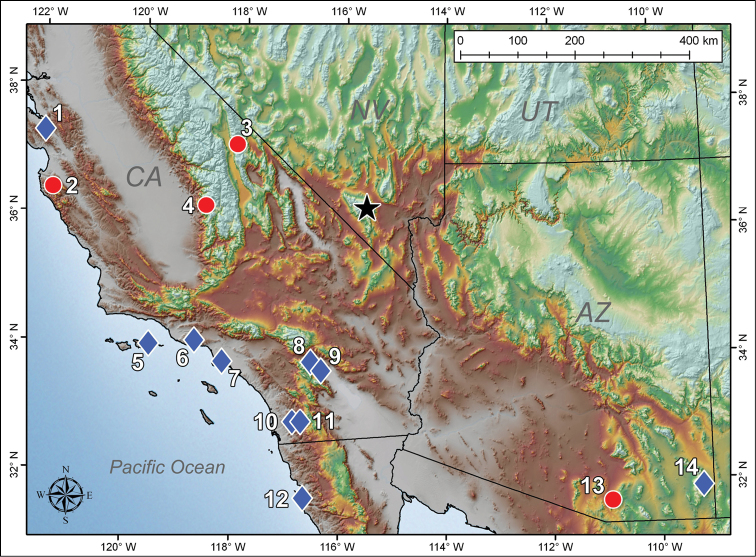
Map depicting the distribution of *Pseudouroctonus peccatum* sp. n. (black star) and type localities of geographically proximate species in genus *Pseudouroctonus* (blue diamonds) and *Uroctonites* (red circles): **1** *Pseudouroctonus glimmei* (Hjelle, 1972) **2** *Uroctonites montereus* (Gertsch & Soleglad, 1972) **3** *Uroctonites giulianii* William & Savary, 1991 **4** *Uroctonites sequoia* (Gertsch & Soleglad, 1972) **5** *Pseudouroctonus minimus thompsoni* (Kraepelin, 1911) **6** *Pseudouroctonus angelenus* (Gertsch & Soleglad, 1972) **7** *Pseudouroctonus minimus minimus* (Kraepelin, 1911) **8** *Pseudouroctonus bogerti* (Gertsch & Soleglad, 1972) **9** *Pseudouroctonus andreas* (Gertsch & Soleglad, 1972) **10** *Pseudouroctonus williamsi* (Gertsch & Soleglad, 1972) **11** *Pseudouroctonus minimus castaneus* (Gertsch & Soleglad, 1972) **12** *Pseudouroctonus rufulus* (Gertsch & Soleglad, 1972) **13** *Uroctonites huachuca* (Gertsch & Soleglad, 1972) **14** *Pseudouroctonus apacheanus* (Gertsch & Soleglad, 1972).

The new species is clearly a member of family Vaejovidae, and is most similar to genera *Pseudouroctonus* and *Uroctonites*, both of which are stenotypic and contain species endemic to sky island ecosystems in southwestern North America. Interestingly, the Spring Mountains are situated in the middle of a substantial gap between the known distributions of these two genera (Fig. 1), so the new species could prove to be a missing link in our understanding of the biogeography of this group (Bryson et al. 2013a) and the southwestern sky islands. Herein, we tentatively place the new species in *Pseudouroctonus*, although we predict that the genus is polyphyletic and in need of a thorough systematic revision.

Since the population at the type locality may have been extirpated during recent fires, we provide DNA barcodes (COI) for two specimens (paratypes) to assist colleagues in their ongoing research on the biogeography and systematics of family Vaejovidae. Given that the species went undetected for so long despite occurring in a populated region near one of the most visited cities in the world, we suspect that similar new species may still await discovery in the more remote and less-explored sky islands of southern Nevada and California.

**Brief taxonomic history.** Of the 22 species and subspecies currently comprising genera *Pseudouroctonus* and *Uroctonites* (including the new species described herein), sixteen were described by Gertsch and Soleglad (1972). At that time, twelve of these species were placed in genus *Uroctonus* (now in family Chactidae) and the other four in genus *Vaejovis*. Stahnke (1974) moved most of the species placed in *Uroctonus* into genus *Vaejovis* and created the new genus *Pseudouroctonus* solely for *Pseudouroctonus reddelli*. Stockwell (1992) reversed most of Stahnke’s taxonomic acts by moving the species Stahnke placed in *Vaejovis* into *Pseudouroctonus*. In their important paper, Williams and Savary (1991) defined the new genus *Uroctonites* comprised of a new species, *Uroctonites giulianii*, and three *Pseudouroctonus* species originally defined by Gertsch and Soleglad (1972). Finally, four other species have now been placed in *Pseudouroctonus*: *Pseudouroctonus minimus minimus* (Kraepelin, 1911), *Pseudouroctonus glimmei* Hjelle, 1972, *Pseudouroctonus sprousei* Francke & Savary, 2006, and *Pseudouroctonus saavasi* Francke (2009). See Soleglad and Fet (2003: 103–104) for a more detailed discussion on the taxonomic history of these interesting scorpions.

## Material and methods

Measurements are as described by Sissom et al. (1990), trichobothrial patterns are as in Vachon (1974) and Soleglad and Fet (2003), and pedipalp finger dentition follows Soleglad and Sissom (2001).

Acronyms of depository.—AMNH, American Museum of Natural History, New York, New York, USA; MRG, personal collection of Matthew R. Graham, Willimantic, Connecticut, USA.

### Molecular analysis

We extracted total genomic DNA from leg tissue from the left side of the two female paratypes using a DNeasy Extraction Kit (Qiagen Inc.). A portion of the cytochrome oxidase subunit I (COI) gene, which is used for barcoding, was amplified with forward primer COI_modF (5’ - ATCATAAGGATATTGGGACTATGT - 3’, Bryson et al. 2013) and reverse primer LE1r (5’ - GTAGCAGCAGTAAARTARGCYCGAGTATC - 3’, Esposito 2011). Double-stranded cycle sequencing was performed using the same primers and the Big Dye Terminator v. 3.1 Cycle 6 Sequencing Kit (Applied Biosystems). The COI sequences were submitted to the Barcode of Life Data system (BOLD, Ratnasingham and Hebert 2007), and GenBank (Accession Nos. KF841448 & KF841449).

## Systematics

### Family Vaejovidae Thorell, 1876
Subfamily Vaejovinae Thorell, 1876
Genus *Pseudouroctonus* Stahnke, 1974

#### Pseudouroctonus peccatum sp. n.

http://zoobank.org/0392FDFD-1BC6-4D7D-B80A-C232C9BDEBCB

http://species-id.net/wiki/Pseudouroctonus_peccatum

Figs 1 –17 ; Table 1 –2

##### Type material.

United States: *Nevada*: female holotype, Kyle Canyon Road, Spring Mountains, 36.2666°N, 115.5988°W, 10 May 2013, A.E. Tate, R.R. Riddle, and M.R. Graham *leg.* (AMNH, MRG1251); paratype, mature male, same location as holotype, A.E. Tate and R.R. Riddle *leg.* (AMNH, MRG1252); paratype, juvenile female, collected with holotype (AMNH, MRG1222); paratype, juvenile female, same location as holotype, 23 April 2013, A.E. Tate, R.R. Riddle, and D.R. Tate *leg.* (AMNH, MRG1221); juvenile female, same location as holotype, 2 July 2013, R.R. Riddle *leg.* (AMNH, MRG1253).

##### Etymology.

The specific epithet is Latin for “sin” in reference to the proximity of the type locality to Las Vegas, which is known informally as “Sin City.”

##### Diagnosis.

Medium to large sized species for the genus, females reaching 50 mm; pectinal tooth counts 13–14 for females and 15 for males. Hemispermatophore lacking secondary lamellar hook; lamellar hook protrudes somewhat from the lamina base creating a modest basal constriction; lamina terminus with a distinct distal crest. Cheliceral movable finger ventral edge with low-profiled pigmented crenulated teeth; fixed finger ventral edge with several small flat pigmented protuberances; movable finger dorsal edge with two subdistal (*sd*) denticles. Chelal movable finger with seven inner denticles (*ID*). Metasomal segment V somewhat stout in adults, length compared to width 1.908 in females and 1.953 in males.

##### Description of holotype.

*Color*: Carapace, trochanter, femur, patella, tergites, and metasoma have a yellow-orange base color with dark brown to black markings along the carinae of the pedipalp and metasoma. Legs are light yellow. Pedipalp chelae are dark brown in color with darker reddish-brown coloration at the anterior portion of the palm where the fixed finger and movable finger meet. Chelicerae are light yellow on proximal half with dark reddish-brown fingers. Vesicle portion of the telson is yellow-orange with a dark reddish-brown to black aculeus. Pectines and genital operculum are light yellow.

*Morphology*: Carapace: trapezoidal with noticeably emarginated anterior margin; surface finely granular with scattered small granules, with larger granules symmetrically flanking the lateral and median eyes; median furrow is slight and traverses length of carapace; ratio of location of median eyes location (from anterior edge)/carapace length = 0.338. Tergites: surface with small granules on distal 1/3–1/2 of tergites I–VI; tergite VII with two pairs of granular lateral carinae, and a slight median hump. Sternites: III–VI smooth to very finely granular and without carinae; VII with granular ventral lateral carinae on posterior 4/5, median carinal pair essentially obsolete. Spiracles: ellipsoid and with median side rotated 40° away from posterior sternite margin. Genital Operculum: sclerites separated on posterior 1/5. Pectines: tooth count 13/13; middle lamellae 8/8; sensorial areas present on all pectine teeth. Metasoma: ratio of segment I length/width 0.91; segment II length/width 1.00; segment III length/width 1.06; segment IV length/width 1.36; segment V length/width 1.91. Segments I–IV: dorsal carinae are moderately denticulate on segments I–IV and have slightly enlarged distal denticles; dorsolateral carinae are moderately crenulate on segment I and moderately denticulate on segments II–IV with enlarged posterior denticles; posterior denticles are largest and most pronounced on dorsolateral carinae IV; ventrolateral carinae are moderately crenulate on segments I–IV; segments II and III have sparse and moderately crenulate intermediary carinae forming an approximately 30° angle with the dorsolateral carinae on the posterior 2/3 on segment II and posterior 1/3 segment III; ventromedian carinae are smooth to obsolete on segment I, and crenulate on segments II–IV; ventrolateral setae 2/2:2/2:2/2:2/2; ventral submedian setae 3/3:3/3:3/3:3/3. Segment V: dorsolateral carinae subtly denticulate; lateral carinae crenulate and obsolete on posterior 1/3; ventrolateral carinae crenulate; ventromedian carinae crenulate; intercarinal spaces finely granular; dorsolateral setation 3/3; lateral setation 3/3; ventrolateral setation 4/4; ventromedian setation 4/4. Telson: smooth to slightly granular with no subaculear tubercule and lacking LAS denticles (Fet et al. 2006). Chelicerae: dorsal edge of fixed finger with four teeth, one distal, one subdistal, one median, and one basal, the latter two denticles formed as a bicuspid; ventral edge with seven low-profile pigmented protuberances, the distal two smaller in size; dorsal edge of movable finger has five teeth total comprised of one distal, two subdistal, one median tooth, and one basal tooth; ventral edge of movable finger has seven small pigmented crenulated teeth; serrula with approximately 23 tines. Pedipalps:trichobothrial pattern type C, orthobothriotaxic: trichobothria *ib-it* positioned on extreme base of fixed finger, *Dt* located considerably basal of palm midpoint, *Db* ventral of digital carina, patellar trichobothria *v_3_* adjacent to *et_3_*; ratio of chela length/width 3.57; femur length/width 2.60; patella length/width 2.79; fixed finger length/carapace length 0.78. Chela: median denticles (MD) of fixed finger aligned and divided into six subrows by five outer denticles (OD); flanked by six inner denticles (ID); movable finger with six subrows of MD, five OD and seven ID. Chela carinae: Digital carina rounded, rough, and somewhat flattened; subdigital essentially obsolete, formed by two granules; dorsosecondary flat, rounded, and roughly textured; dorsomarginal very rounded, with large scattered granules; dorsointernal rounded, with scattered granules; ventroexternal rounded, roughly textured, terminating at external condyle of movable finger; ventromedian flat, essentially obsolete; ventrointernal rounded, with small granules scattered distally; external carina very rounded, roughly textured. Femur: dorsointernal, and ventrointernal carinae denticulate and black in color, ventroexternal and dorsoexternal carinae denticulate with small granules throughout, internal surface has 6 prominent dentate granules. Patella:dorsointernal carinae are denticulate, dorsoexternal carinae are mildly crenate, ventral internal carinae are dentate, ventroexternal carinae are moderately denticulate, internal surface has 11 prominent granules. Legs: Ventral surface of tarsus with single median row of 7–17 spinules terminating distally with one to two pairs of spinules. The terminal spinules are slightly stouter than the those comprising the median row. Flanking setal pairs are essentially absent in legs I–II, with 2–3 irregularly positioned and sized pairs on legs III–IV. Basitarsus spinule rows are limited, 2 rows on leg I, 1 row on leg II, 1 weak row on III, and absent on IV. Basitarsus populated with large irregularly placed darkly pigmented setae. Hemispermatophore: Left hemispermatophore is 5.7 mm in length; lamina length 3.71, lamellar hook length 1.03, and trough different (vertical distance between ventral and dorsal troughs) 0.55. Lamellar edges sub-parallel, except for slight expansion on mid-area of the internal edge; terminus blunted with a distinct distal crest on the dorsal side. Lamellar hook extends somewhat from lamina base, is distinctly bifurcated, and is formed entirely from the dorsal trough. A modest basal constriction and a deep truncal flexure are present. Sclerotized mating plugs with smooth barbs were extracted from the ventrointernal aspect of both hemispermatophore median areas.

**Figure 2–3.**
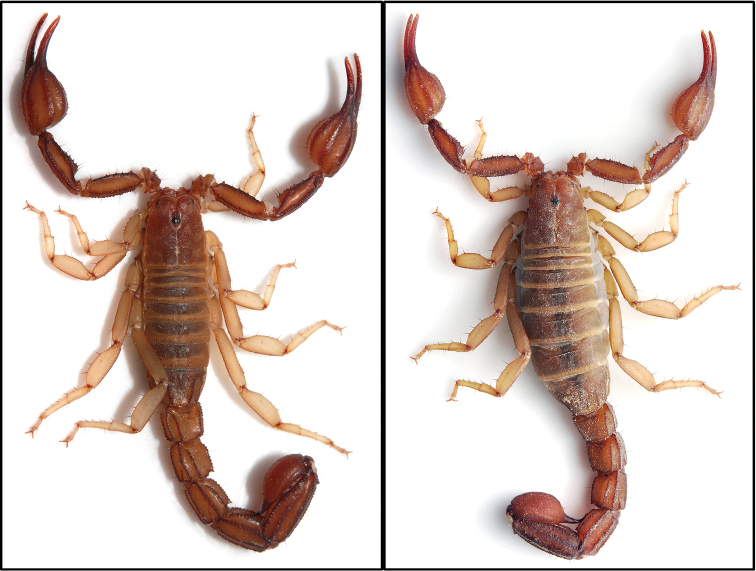
Dorsal view of male (**2**) and female (**3**) *Pseudouroctonus peccatum* sp. n. *in vivo*.

**Figures 4–15.**
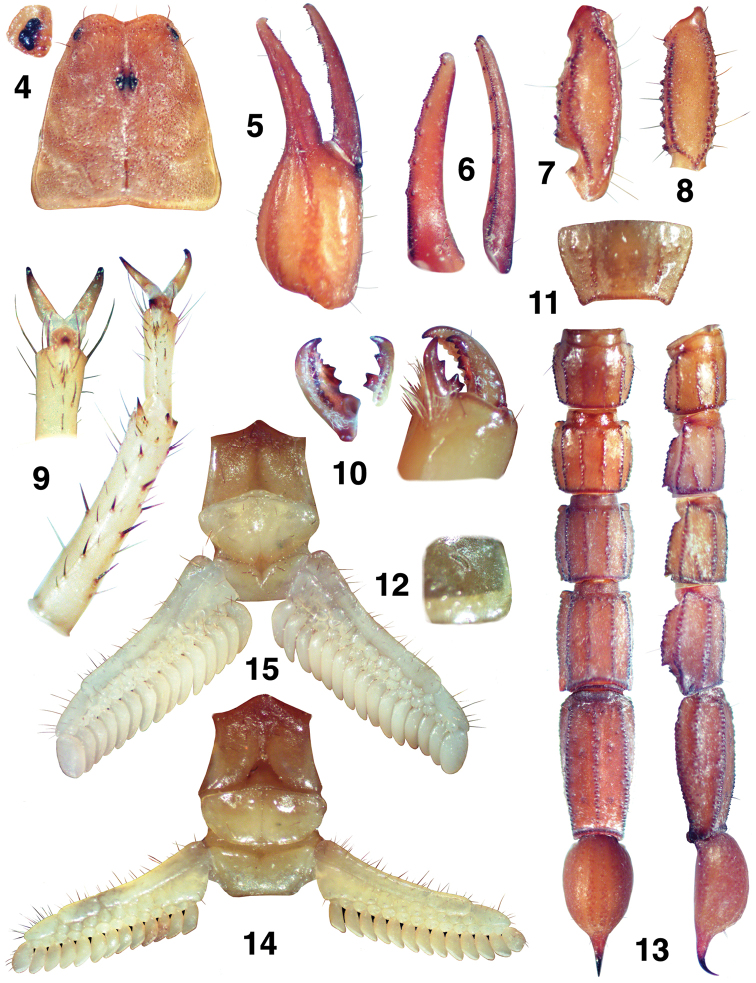
*Pseudouroctonus peccatum* sp. n., Spring Mountains, Clark Co., Nevada, USA. Female holotype. **4** Carapace and close-up of lateral eyes. **5–8** Right pedipalp **5** Chela, external view **6** Chelal finger dentition, fixed and movable fingers **7** Patella, dorsal view **8** Femur, dorsal view **9** Right leg II, tarsus close-up, partial ventral view. Right leg III, tarsus and basitarsus, ventral view **10** Cheliceral movable and fixed fingers, ventral view. Chelicera, dorsal view **11** Sternite VII **12** Left stigma I **13** Metasoma and telson, ventral and lateral views **14** Sternopectinal area **15** Male paratype, sternopectinal area.

**Figure 16.**
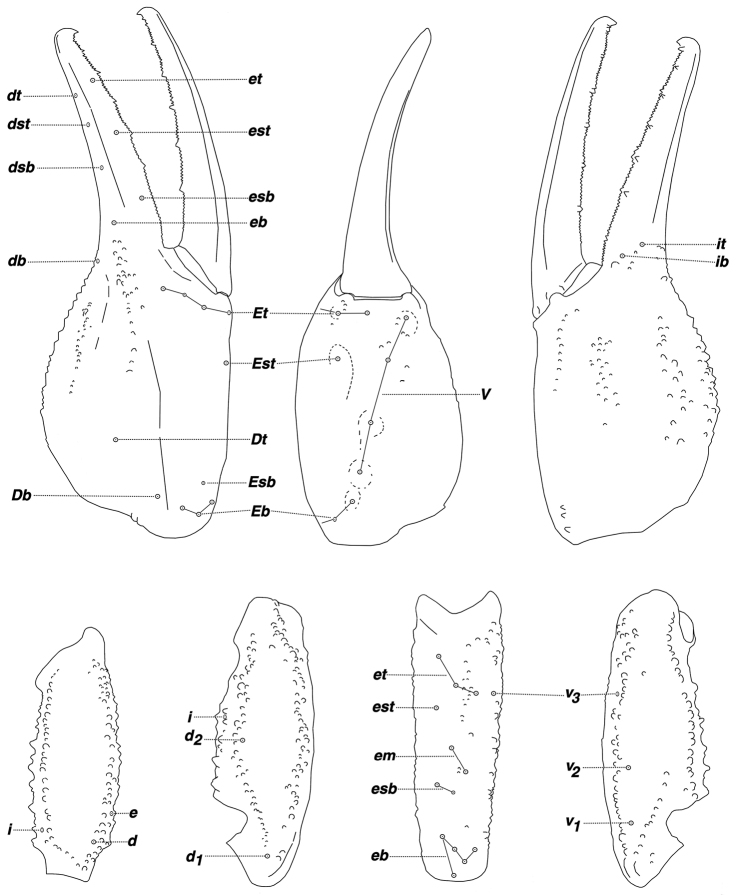
*Pseudouroctonus peccatum* sp. n. female holotype, Spring Mountains, Clark Co., Nevada, USA. Trichobothrial pattern.

**Figure 17.**
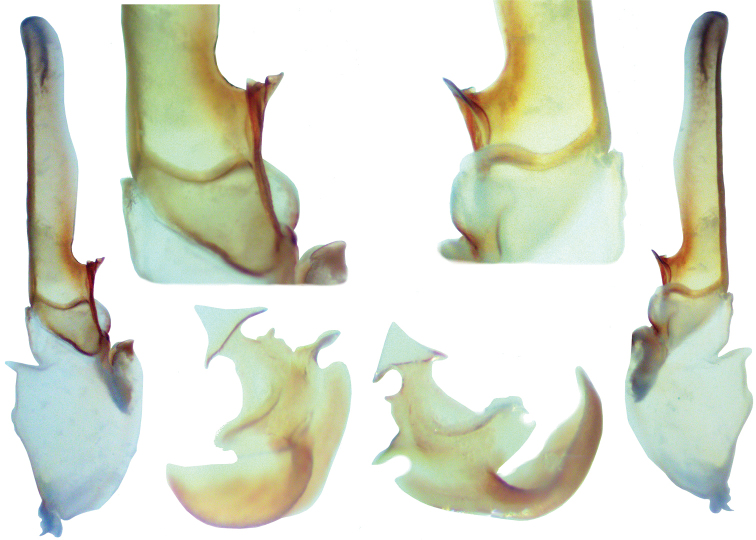
*Pseudouroctonus peccatum* sp. n., male paratype, Spring Mountains, Clark Co., Nevada, USA. Left hemispermatophore and mating plug (both reversed, photographed submerged in alcohol). **Outside** hemispermatophore, dorsal and ventral views. **Top** closeup of median area, dorsal and ventral views. Note, due to the translucency of the hemispermatophore,the sclerotized ventral trough is partially visible from the dorsal side. **Bottom** mating plug. dorsal and ventral views. Note, the sclerotized edge of the mating plug barb is located on the dorsal surface and is partially visible from the ventral side due to the translucency of the plug.

**Figure 18.**
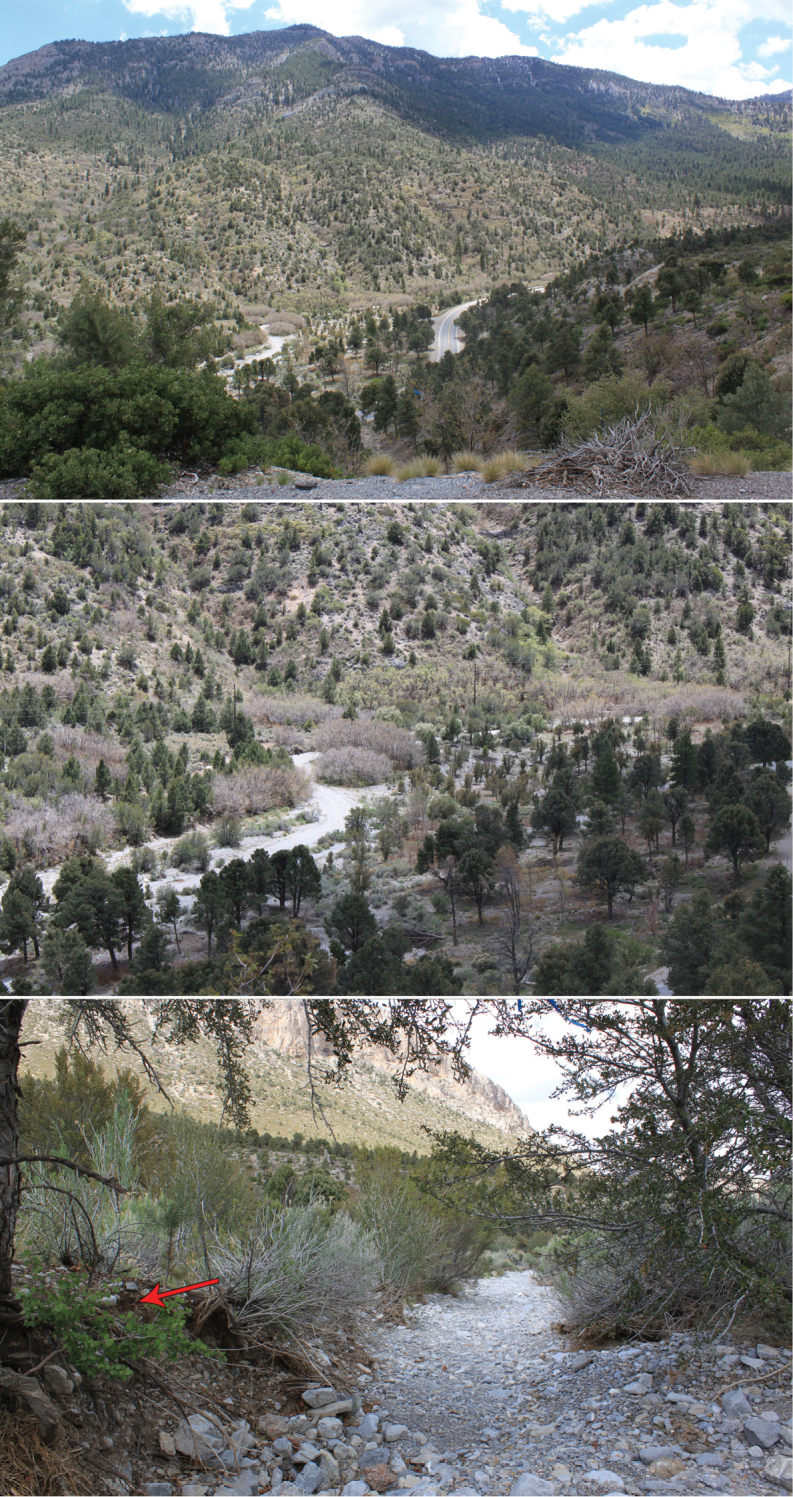
Type locality of *Pseudouroctonus peccatum* sp. n. on the eastern slope of the Spring Mountains, Nevada. Red arrow indicates where the holotype was discovered.

**Table 1.** Measurements (in millimeters) of all known specimens of *Pseudouroctonus peccatum*.

Scorpion ID	Holotype MRG1251	Female MRG1221	Female MRG1222	Male MRG1252	Female MRG1253
Total Length	52.45 mm	32.37 mm	27.71 mm	36.12 mm	39.83 mm
Carapace L	5.59 mm	4.37 mm	3.66 mm	4.53 mm	4.90 mm
Mesosoma L	19.89 mm	8.30 mm	8.67 mm	9.79 mm	13.94 mm
Metasoma L	20.8 mm	15.00 mm	11.70 mm	17.03 mm	16.36 mm
MET I L	3.15 mm	2.43 mm	1.98 mm	2.66 mm	2.53 mm
MET I W	3.45 mm	2.55 mm	2.12 mm	2.63 mm	2.81 mm
MET I D	2.65 mm	1.98 mm	1.62 mm	1.99 mm	2.20 mm
MET II L	3.43 mm	2.40 mm	1.79 mm	2.84 mm	2.69 mm
MET II W	3.42 mm	2.55 mm	2.12 mm	2.65 mm	2.79 mm
MET II D	2.91 mm	1.98 mm	1.55 mm	2.03 mm	2.31 mm
MET III L	3.59 mm	2.52 mm	1.90 mm	3.00 mm	2.82 mm
MET III W	3.39 mm	2.55 mm	2.06 mm	2.69 mm	2.69 mm
MET III D	2.62 mm	1.95 mm	1.62 mm	2.07 mm	2.22 mm
MET IV L	4.41 mm	3.06 mm	2.29 mm	3.61 mm	3.20 mm
MET IV W	3.25 mm	2.48 mm	1.98 mm	2.51 mm	2.63 mm
MET IV D	2.57 mm	1.95 mm	1.59 mm	2.15 mm	2.52 mm
MET V L	6.22 mm	4.59 mm	3.74 mm	4.98 mm	5.12 mm
MET V W	3.26 mm	2.48 mm	2.01 mm	2.55 mm	2.59 mm
MET V D	2.47 mm	1.77 mm	1.54 mm	1.95 mm	2.17 mm
Telson L	6.17 mm	4.47 mm	3.68 mm	4.77 mm	4.63 mm
Vesicle L	4.21 mm	3.02 mm	2.30 mm	3.39 mm	3.49 mm
Vesicle W	3.13 mm	2.31 mm	1.70 mm	2.33 mm	2.19 m
Vesicle D	2.41 mm	1.68 mm	1.49 mm	1.67 mm	2.62 mm
Aculeus L	1.96 mm	1.45 mm	1.38 mm	1.38 mm	1.14 mm
Pedipalp L	18.79 mm	15.09 mm	11.80 mm	16.08 mm	16.44 mm
Femur L	4.71 mm	3.74 mm	2.96 mm	3.88 mm	4.30 mm
Femur W	1.81 mm	1.26 mm	1.11 mm	1.39 mm	1.41 mm
Femur D	1.24 mm	0.95 mm	0.82 mm	1.00 mm	0.95 mm
Patella L	5.02 mm	3.80 mm	3.40 mm	4.05 mm	4.41 mm
Patella W	1.80 mm	1.43 mm	1.19 mm	1.42 mm	1.59 mm
Patella D	1.71 mm	1.38 mm	0.92 mm	1.34 mm	1.38 mm
Chela L	9.06 mm	7.55 mm	5.27 mm	8.15 mm	7.73 mm
Palm W	2.54 mm	1.81 mm	1.26 mm	2.10 mm	1.49 mm
Palm L	4.84 mm	2.85 mm	2.46 mm	3.79 mm	3.38 mm
Chela D	3.31 mm	2.23 mm	1.44 mm	2.84 mm	2.65 mm
MF L	5.55 mm	4.05 mm	3.33 mm	4.36 mm	4.35 mm
FF L	4.37 mm	3.32 mm	2.94 mm	3.25 mm	3.65 mm
Cara to eyes	1.89 mm	1.65 mm	1.20 mm	1.37 mm	1.67 mm
**Pectine count**	13/13	14/14	13/13	15/15	14/14

**Table 2.** Matrix showing character set that distinguishes *Pseudouroctonus peccatum* sp. n. from the other seventeen species in the genus. If a species differs significantly for a given data character, its state is shaded. Note, except for *Pseudouroctonus peccatum* sp. n., most of the data contained in this table is based on published information (see reference list). *ID* = inner denticle.

	Hemispermatophore	Cheliceral<br/> Dentition	Chelal Movable Finger *ID*	Pectinal Tooth *Counts*	Metasoma Seg V (Length/Width)
***Pseudouroctonus peccatum***	Distal crest present on lamina terminus; secondary lamellar hook absent	*va* on MF and FF crenulated; two subdistal<br/> denticles	Seven *ID*	15 ♂13–14 ♀	1.953 ♂1.908 ♀
***Pseudouroctonus andreas***	Distal crest absent	smooth	Six *ID*	smaller<br/> 9–10 ♂ 7–9 ♀	-<br/> 1.933 ♂2.000 ♀
***Pseudouroctonus angelenus***	Distal crest absent; secondary lamellar hook present	smooth	Seven *ID*	smaller<br/> 11 ♂ ? ♀	thinner<br/> 3.059 ♂? ♀
***Pseudouroctonus apacheanus***	?	smooth	Seven *ID*	smaller<br/> 10–11 ♂ 9–10♀	-<br/> 2.412 ♂2.176 ♀
***Pseudouroctonus bogerti***	Distal crest absent; secondary lamellar hook present	smooth	Seven *ID*	-? ♂ 12 ♀	thinner<br/> 3.444 ♂3.091 ♀
***Pseudouroctonus cazieri***	Distal crest absent	*va* on MF and FF toothed	Six *ID*	-<br/> 13 ♂ 11–12 ♀	-<br/> 2.150 ♂2.217 ♀
***Pseudouroctonus chicano***	?	smooth	Seven *ID*	smaller<br/> ? ♂ 9♀	-<br/> ? ♂2.067 ♀
***Pseudouroctonus glimmei***	Distal crest absent; secondary lamellar hook present	smooth	Seven *ID*	smaller<br/> 10–12 ♂ 9–11♀	thinner<br/> 2.600 ♂2.609 ♀
***Pseudouroctonus iviei***	Distal crest absent; secondary lamellar hook present	smooth	Seven *ID*	smaller<br/> 11 ♂ 10♀	-<br/> 2.191 ♂2.304 ♀
***Pseudouroctonus lindsayi***	Distal crest absent	smooth	Six *ID*	-<br/> 14 ♂ 12 ♀	-<br/> 2.286 ♂2.286 ♀
***Pseudouroctonus minimus castaneus***	Distal crest absent	smooth	Seven *ID*	smaller<br/> 10–12 ♂ 10♀	wider<br/> 1.402 ♂1.364 ♀
***Pseudouroctonus minimus minimus***	Distal crest absent	smooth	Six *ID*	smaller<br/> 10–11 ♂ 9–10♀	wider<br/> 1.650 ♂1.565 ♀
***Pseudouroctonus minimus thompsoni***	?	smooth	Seven *ID*	smaller<br/> 10–11 ♂ 10♀	wider<br/> 1.667 ♂1.538 ♀
***Pseudouroctonus reddelli***	Distal crest absent	*va* on MF and FF toothed	Seven *ID*	larger<br/> 18–19 ♂ 15–16♀	thinner<br/> 3.036 ♂2.871 ♀
***Pseudouroctonus rufulus***	Distal crest absent	smooth	Seven *ID*	smaller<br/> 11 ♂ 9♀	-<br/> 2.400 ♂2.176 ♀
***Pseudouroctonus saavasi***	Distal crest absent	Smooth, one subdistal (*sd*)	Seven *ID*	smaller<br/> 10–11 ♂ 9♀	thinner<br/> 2.810 ♂2.563 ♀
***Pseudouroctonus sprousei***	Distal crest absent	*va* on MF and FF toothed	Seven *ID*	larger<br/> 17 ♂ ?♀	thinner<br/> 4.214 ♂? ♀
***Pseudouroctonus williamsi***	Distal crest absent; secondary lamellar hook present	smooth	Seven *ID*	-<br/> 13 ♂ 11–12 ♀	thinner<br/> 3.400 ♂3.400 ♀

##### DNA barcodes

(COI) – MRG1221 (paratype, GenBank no. KF841448):

CTCTAAGTTTAATGATTCGTGCGGAAATTGGTAGACCTGGGTCTTTTATCGGGGATGATCAAATTTATAATGTTGTGGTTACTGCTCATGCTTTTGTCATAATTTTTTTTATGGTTATGCCAATTATGATTGGGGGTTTTGGTAATTGGTTAGTTCCTTTGATGTTAGGTGCTCCTGATATGGCTTTTCCTCGTTTAAATAATATAAGATTTTGATTATTACCACCTGCATTTTTTATGCTTTTAGGTTCGGCATCGTTGGAAAGAGGGGCAGGTACAGGCTGAACTGTGTACCCTCCTCTTTCCTCATATATGTTTCATTCTGGGGGGTCTGTTGATATGACTATTTTTTCGTTACATTTGGCTGGTGTTTCTTCAATTTTAGGAGCTATTAATTTTATTACTACTATTTTGAATATGCGAAGATCTGGAATATTGTTGGAGCGTGTGCCTTTATTTGTATGGTCTGTTAAGATTACTGCTATTCTTCTGTTGTTGTCTCTTCCAGTTCTTGCGGGTGCAATTACTATGCTATTAACAGATCGAAATTTTAATACTTCTTTTTTTGATCCAGCGGGTGGAGGGGATCCTATTTTGTACCAACATTTATTTTGATTTTTTGGTCATCCTGAGGTTTATATTTTAATTCTTCCGGGATTTGGAATGATTTCTCATATTATTAGTCATCATACTGGGAAGAGGGAACCTTTCGGAGCTTTAGGAATGATTTATGCTATGGTGGCTATTGGGTTTTTGGGTTTTGTGGTTTG.

MRG1222 (paratype, GenBank no. KF841449):

CTCTAAGTTTAATGATTCGTGCGGAAATTGGTAGACCTGGGTCTTTTATCGGGGATGATCAAATTTATAATGTTGTGGTTACTGCTCATGCTTTTGTCATAATTTTTTTTATGGTTATGCCAATTATGATTGGGGGTTTTGGTAATTGGTTAGTTCCTTTGATGTTAGGTGCTCCTGATATGGCTTTTCCTCGTTTAAATAATATAAGATTTTGATTATTACCACCTGCATTTTTTATGCTTTTAGGTTCGGCATCGTTGGAAAGAGGGGCAGGTACAGGCTGAACTGTGTACCCTCCTCTTTCCTCATATATGTTTCATTCTGGGGGGTCTGTTGATATGACTATTTTTTCGTTACATTTGGCTGGTGTTTCTTCAATTTTAGGAGCTATTAATTTTATTACTACTATTTTGAATATGCGAAGATCTGGAATATTGTTGGAGCGTGTGCCTTTATTTGTATGGTCTGTTAAGATTACTGCTATTCTTCTGTTGTTGTCTCTTCCAGTTCTTGCGGGTGCAATTACTATACTATTAACAGATCGAAATTTTAATACTTCTTTTTTTGATCCAGCGGGTGGAGGGGATCCTATTTTGTACCAACATTTATTTTGATTTTTTGGTCATCCTGAGGTTTATATTTTAATTCTTCCGGGATTTGGAATGATTTCTCATATTATTAGTCATCATACTGGGAAGAGGGAACCTTTCGGAGCTTTAGGAATGATTTATGCTATGGTGGCTATTGGGTTTTTGGGTTTTGTGGTTTG.

## Discussion

The adult male and female can be differentiated by the larger pectinal tooth counts in the male (15 versus 13–14), the completely separated genital operculum in the male and the presence of genital papillae (in the female the sclerites are only separated on the posterior one-quarter and papillae are absent). The adult female is larger in size, 52 mm versus 36 mm. The metasoma of the male is slightly thinner than in the female, exhibiting the following percentage differences for all five segments when the segment’s length is compared to its width (2.4–10.8%).

On the dorsal edge of the right cheliceral movable finger of the female holotype, a small bifurcation is present on the distal aspect of the median (*m*) denticle (see Fig. 10). This bifurcation is not present on the left chelicerae, so we consider it an anomaly.

## Distribution

Known only from the type locality in the Spring Mountains of Southern Nevada where it was collected at an elevation of 2,103 m.

### Comparison to similar species

Soleglad and Fet (2008: fig. 196) considered *Uroctonites* and *Pseudouroctonus* to form a distinct clade within the vaejovid subfamily Vaejovinae. Pending results from detailed morphological and molecular analyses, we place *Pseudouroctonus peccatum* sp. n. in this group based on the following characters: (1) the chelae are thick with swollen palms that are somewhat flat in appearance due to the weak to obsolete development of the dorsosecondary and ventromedian carinae; (2) the carapace exhibits a conspicuous anterior emargination; (3) the median eyes are reduced in size and located considerably anterior of the carapace midpoint; (4) the ventral edges of the cheliceral movable finger and fixed finger are equipped with small crenulations and protuberances, respectively; these two characters are found in many species of this group and likewise generally absent in the other genera comprising the subfamily (*Vaejovis* and *Franckeus*); (5) the serrulae are well-developed; (6) the pectinal tooth counts as compared to the scorpion’s adult size are relatively small.

Within the clade, *Pseudouroctonus peccatum* clearly has a closer affinity to *Pseudouroctonus* than *Uroctonites*. The sides of the hemispermatophore lamina are sub-parallel (not tapered), the terminus is truncated (not pointed); lamellar hook is located distal of dorsal trough (not adjacent to), and the terminus is bifurcated (not intact). The mating plug is sclerotized (not partially gelatinous). Ventral setal pairs of the leg tarsus are irregularly positioned and sized, and of medium development (not aligned, the same size, and stout). Although the relative pectinal tooth counts in this species are somewhat small, they are not as small as seen in *Uroctonites*. For example, *Pseudouroctonus peccatum* and *Uroctonites huachuca* are similarly sized species, but *Pseudouroctonus peccatum* has 67% (females) and 76% (males) more teeth than *Uroctonites huachuca*. Pectinal tooth counts in the four species of *Uroctonites* range 7–10 in females and 8–10 in males.

*Pseudouroctonus* is comprised of eighteen species (including *Pseudouroctonus peccatum*). The new species can be separated from the other species based on the structure of its hemispermatophore, cheliceral and chelal dentition, pectinal tooth counts, and morphometrics of the fifth metasomal segment (see Table 2 for character comparisons across all species). Before addressing each of the seventeen species, we must point out that *Pseudouroctonus peccatum* has a distal crest on the hemispermatophore lamina terminus and its lamellar hook is somewhat removed from the lamina base, providing a small basal constriction. Both of these characters are unknown from any of the hemispermatophores so far reported for this genus [note: we have information on the hemispermatophore for fifteen *Pseudouroctonus* species, only three are unknown. See Williams and Savary (1991: figs 21–29), Francke and Savary (2006: figs 15–21), Soleglad and Fet (2008: figs 67–69), and Francke (2009: figs 2–4)]. Although it is clear, based on Table 2, that more than one diagnostic character is available for separating *Pseudouroctonus peccatum* from the other species, we will restrict our discussion to discrete characters thus minimizing the dependence on meristic and morphometric data. *Pseudouroctonus peccatum* does not have a secondary hook on the hemispermatophore as found in *Pseudouroctonus williamsi*, *Pseudouroctonus angelenus*, *Pseudouroctonus bogerti*, *Pseudouroctonus glimmei*, and *Pseudouroctonus iviei*. Although *Pseudouroctonus peccatum* is equipped with crenulated denticles on the ventral edge of the cheliceral movable finger, as well as small protuberances on the venter of the fixed finger, they are not enlarged and tooth-like as found in *Pseudouroctonus reddelli*, *Pseudouroctonus sprousei*, and *Pseudouroctonus cazieri*. The lamellar hook on the hemispermatophoreof *Pseudouroctonus peccatum* is distal from the dorsal trough, whereas in *Pseudouroctonus lindsayi* it is adjacent. *Pseudouroctonus peccatum* has two subdistal (*sd*) denticles on the cheliceral movable finger dorsal edge, whereas the cave adapted species *Pseudouroctonus saavasi* has only one. *Pseudouroctonus peccatum* has seven inner denticles (*ID*) on the chelal movable finger, whereas *Pseudouroctonus andreas* has only six. As expected given its somewhat large size, *Pseudouroctonus peccatum* has a relatively large pectinal tooth count (i.e., 15 male and 13–14 female), separating it from *Pseudouroctonus chicano*, *Pseudouroctonus apacheanus*, and *Pseudouroctonus rufulus* which range from 10–11 in males and 9–10 in females. Although *Pseudouroctonus peccatum* has a relatively stout metasomal segment V, it is not as robust as that found in *Pseudouroctonus minimus minimus*, *Pseudouroctonus minimus castaneus*, and *Pseudouroctonus minimus thompsoni*. Segment length/width ratios are 1.908 for the female holotype and 1.953 for the male paratype, compared to 1.364–1.565 in females and 1.402–1.667 males for the *Pseudouroctonus minimus* subspecies.

Interestingly, *Pseudouroctonus peccatum* was not found within the known range of any of the *Pseudouroctonus* species, and was discovered closer to two species of *Uroctonites*, *Uroctonites giulianii* and *Uroctonites sequoia*; the distances between the type localities are roughly 258 and 282 km, respectively. The most geographically proximate species of *Pseudouroctonus* are all found in southern California: all three subspecies of *Pseudouroctonus minimus*, *Pseudouroctonus angelenus*, *Pseudouroctonus bogerti*, *Pseudouroctonus williamsi*, and *Pseudouroctonus andreas*. The distance from these type localities ranges 290–426 km. Based on Table 2, *Pseudouroctonus peccatum* appears more closely related to *Pseudouroctonus andreas*, the smallest species in the genus, and the three subspecies of *Pseudouroctonus minimus*. The other *Pseudouroctonus* spp. (*Pseudouroctonus angelenus*, *Pseudouroctonus bogerti*, and *Pseudouroctonus williamsi*) exhibit considerable differences in the hemispematophore structure.

## Supplementary Material

XML Treatment for Pseudouroctonus peccatum
